# Supplementary material for: Deletion of wfs1 Impairs Oligodendrocyte Precursor Cells Dorsal Distribution and Myelination Through the wfs1-hmgcs1 Axis in Zebrafish
Source: Biology (Basel). 2026 Aug 16;15(16):1402. doi: 10.3390/biology15161402 (PMC13510151; doi:10.3390/biology15161402)
Supplement: Supplementary file 1 [file biology-15-01402-s001.zip › File S1-Transcriptome sequencing data and differentially expressed genes (DEGs) in wfs1-- zebrafish at 5 dpf/2.KEGG/wfs1KOvsWT/UP/src/dre00561.html]

dre00561


- dre:322481

- Up regulated genes

322481(1.25923363606388)
- dre:565117

- Up regulated genes

565117(2.67130454108397)

- dre:799099

- Up regulated genes

799099\_1(1.05373800076525)

- dre:322481

- Up regulated genes

322481(1.25923363606388)
- dre:565117

- Up regulated genes

565117(2.67130454108397)

- dre:572841

- Up regulated genes

572841(1.7118841137558)

- dre:368239

- Up regulated genes

368239(1.14034759711527)

- dre:445326

- Up regulated genes

445326\_1(2.09813524726464)

- dre:450083

- Up regulated genes

450083(2.11033262833863)

- dre:724010

- Up regulated genes

724010\_1(10.241655134474)

Close
